# Impact of Sleep–Wake-Associated Neuromodulators and Repetitive Low-Frequency Stimulation on Human iPSC-Derived Neurons

**DOI:** 10.3389/fnins.2019.00554

**Published:** 2019-05-29

**Authors:** Remi Yokoi, Miho Okabe, Naoki Matsuda, Aoi Odawara, Akihiro Karashima, Ikuro Suzuki

**Affiliations:** Department of Electronics, Graduate School of Engineering, Tohoku Institute of Technology, Sendai, Japan

**Keywords:** human iPSC-derived neuron, micro-electrode array, circadian rhythms, neurotransmitters, low-frequency stimulation, wake, non-REM sleep, long-term depression

## Abstract

The cross-regional neurons in the brainstem, hypothalamus, and thalamus regulate the central nervous system, including the cerebral cortex, in a sleep–wake cycle-dependent manner. A characteristic brain wave, called slow wave, of about 1 Hz is observed during non-REM sleep, and the sleep homeostasis hypothesis proposes that the synaptic connection of a neural network is weakened during sleep. In the present study, *in vitro* human induced pluripotent stem cell (iPSC)-derived neurons, we investigated the responses to the neuromodulator known to be involved in sleep–wake regulation. We also determined whether long-term depression (LTD)-like phenomena could be induced by 1 Hz low-frequency stimulation (LFS), which is within the range of the non-REM sleep slow wave. A dose-dependent increase was observed in the number of synchronized burst firings (SBFs) when 0.1–1000 nM of serotonin, acetylcholine, histamine, orexin, or noradrenaline, all with increased extracellular levels during wakefulness, was administered to hiPSC-derived dopaminergic (DA) neurons. The number of SBFs repeatedly increased up to 5 h after 100 nM serotonin administration, inducing a 24-h rhythm cycle. Next, in human iPSC-derived glutamate neurons, 1 Hz LFS was administered four times for 15 min every 90 min. A significant reduction in both the number of firings and SBFs was observed in the 15 min immediately after LFS. Decreased frequency of spontaneous activity and recovery over time were repeatedly observed. Furthermore, we found that LFS attenuates synaptic connections, and particularly attenuates the strong connections in the neuronal network, and does not cause uniform attenuation. These results suggest sleep–wake states can be mimicked by cyclic neuromodulator administration and show that LTD-like phenomena can be induced by LFS *in vitro* human iPSC-derived neurons. These results could be applied in studies on the mechanism of slow waves during sleep or in an *in vitro* drug efficacy evaluation depending on sleep–wake state.

## Introduction

One of ubiquitous phenomenon in living organisms is sleep. Sleep studies have been conducted using various approaches, which have elucidated many of the mysteries inherent in “how we sleep.” In animal experiments recording nerve activity during sleep, it has been found that wide modulating system neurons present in the hypothalamus and brain stem alter activity either simultaneously with or prior to the transition between the sleep and waking states. For example, it has been reported that acetylcholine neurons in the pontine tegmentum and anterior hypothalamus are either REM-on neurons that become selectively active during REM sleep or WR neurons that become active while awake (W) or during REM sleep (R) (Takahashi et al., 2009; Sakai, 2012). In addition, the noradrenaline neurons of the locus coeruleus, serotonin neurons of the raphe nuclei, histamine neurons of the tuberomammillary nuclei, and orexin neurons in the posterior hypothalamus are referred to as W-on or REM-off neurons, because they become active while awake but cease activity during REM sleep (Mcginty and Harper, 1976; Takahashi et al., 2006, 2008, 2009, 2010; Sakai, 2012). Recently developed optical genetics (optogenetics) technologies have enabled the manipulation of specific types of neural activity using light. The selective activation of noradrenaline, orexin, or acetylcholinergic neurons using this technique reportedly shortens the duration of non-REM sleep and increases the duration of wake time or the duration of REM sleep when the cerebral cortex is as strongly activated as it is in an awake state (Adamantidis et al., 2007; Carter et al., 2010; Han et al., 2014; Van Dort et al., 2015). Therefore, it is believed that neurons of the wide modulating system play an important role in sleep–wake control.

The question of why we sleep is also one of the most interesting mysteries in biology. If sleep duration is shortened during the period of brain development, the development of the brain is delayed (Frank et al., 2001). The brains of international flight crews experiencing disturbed sleep rhythm have also been observed to significantly shrink (Cho, 2001). Hence, it is believed that sleep plays an important role in the development, maintenance, and organization of cerebral neural circuits. In Tononi and Cirelli (2003) published a “sleep homeostasis hypothesis” that attempts to explain the neural basis for the above findings. This hypothesis suggests that connections between neurons (synaptic connection strength), which is enhanced during the awakened state, is attenuated and kept within a specific range during sleep. Although there was little evidence in support of this hypothesis when it was published, various subsequent human and animal experiments have shown that synaptic strength is increased during the awakened state and attenuated as described below (Shepherd, 2012; Cirelli, 2013). Studies on the relationship between molecular changes and sleep–walking have reported that sleep–wake rhythm affects the number and phosphorylation levels of glutamate AMPA receptors. For example, in the cerebral cortex and hippocampus, AMPA receptors, including the GluR1 subunit, which play an important role in synaptic long-term potentiation and are significantly increased after awakening compared with after sleep (Vyazovskiy et al., 2008; Lante et al., 2011). Furthermore, the dephosphorylation level of GluR1 Ser845, which is associated with synaptic long-term depression (LTD) as observed in synaptoneurosome, is reportedly higher after sleep (Vyazovskiy et al., 2008). Several studies have investigated the relationship between sleep–wake and synaptic strength using electrophysiological experiments. For example, *in vivo* experiments, the evoked potential by electrical stimulation in the cortex reportedly increases after awakening and decreases after sleep (Vyazovskiy et al., 2008). In addition, experiments in humans involving the measurement of the evoked potential by transcranial magnetic stimulation have reported that the response increases during awakening and sleeplessness and decreases after sleep (Huber et al., 2013). These studies suggested that synaptic connections are attenuated during sleep in humans and animals. Although the entire mechanism of the attenuation of the synapse binding during sleep has not yet been elucidated, slow waves appearing during non-REM sleep may play an important role (Tononi and Cirelli, 2006). One reason underlying this conclusion is that the slow wave cycle is similar to the low-frequency electrical stimulation (LFS) cycle, which induces synaptic LTD (Kemp and Bashir, 2001).

The discovery of responses to neuromodulators related to sleep–wake regulation and the phenomena related to LTD during sleep *in vitro* human-derived neurons will aid the elucidation of the mechanism of neural network dynamics that occur during sleep–awakening and research of diseases, such as several sleep disorders. In addition, it will be possible to evaluate safety assessment, such as seizure liability of new drugs, depending on sleep-wake rhythm. iPSC-derived neurons (Takahashi and Yamanaka, 2006) are considered suitable to be used as *in vitro* evaluation samples because they can be induced to differentiate into specific neuronal cells in the human brain. For example, the cerebral cortex (Shi et al., 2012) and midbrain dopamine neurons (Studer, 2012) have been created from human iPSCs.

The micro-electrode array (MEA) measurement is one of the most effective methods for evaluating the electrical activity of *in vitro* human iPSC-derived neurons, and it has been recently used to assess drug efficacy (Odawara et al., 2014, 2016, 2018; Ishii et al., 2017; Kasteel and Westerink, 2017; Grainger et al., 2018; Kreir et al., 2018; Ojima and Miyamoto, 2018). In addition, we have developed methods to assess the seizure liability of drugs using the MEA method in cultured human iPSC-derived neurons (Matsuda et al., 2018; Odawara et al., 2018). We have detected seizure-like activities by the administration of convulsants and identified differences based on the drugs’ mechanism of action (Odawara et al., 2014, 2016, 2018; Matsuda et al., 2018). The MEA measurement method is also suitable in the study of circadian rhythm because it can measure *in vitro* neural network activity for a long time (Honma et al., 2004; Enoki et al., 2017a,b).

In this study, we focused on neural activity during sleep–wakening and attempted to determine whether responses to neuromodulators associated with sleep–wake states could be detected, and whether attenuation of network activity during sleep could be detected by LFS *in vitro* human iPSC-derived neurons. Serotonin, acetylcholine, histamine, orexin, and noradrenaline, all of which are neurotransmitters released from neurons playing important roles in sleep–wake regulation, were administered and short- and long-term changes in neural network activity were measured in dopaminergic (DA) neurons. In addition, we constructed a neural network with a high percentage of glutamatergic neurons rich in glutamate receptors, which are known to be involved in the generation of LTD. An electrical stimulus of 1 Hz was administered to this neural network, and we verified whether LTD-like phenomena can be induced.

## Materials and Methods

### Culture of hiPSC-Derived Neurons

Human iPSC-derived DA neurons [iCell DopaNeurons, DNC-301-030-001, FUJIFILM Cellular Dynamics, Inc (FCDI)] were cultured at 8.0 × 10^5^ cells/cm^2^ on 16-channels per well across 4-well MEA plates (MED-P5NF30, Alpha Med Scientific) and 24-well MEA plate (MED-Q2430M, Alpha Med Scientific Inc.) coated with Polyethyleneimine (Sigma) and Laminin-511 (Nippi). Two vials of DA neurons were cultured on different days, respectively. Human iPSC-derived astrocytes (iCell Astrocyte, ASC-100-020-001-PT, FCDI) were seeded at 5.4 × 10^4^ cells per well. After 1 day, the medium was replaced with BrainPhys Neuronal Medium (STEMCELL technologies) with iCell DopaNeurons Medium Supplement (FCDI), Laminin (Sigma), N2 Supplement (STEMCELL technologies), and 100 U/mL penicillin/streptomycin (168-23191, Wako). Human iPSC-derived Glutamatergic neuron (iCell GlutaNeurons, R1061, FCDI) and astrocytes (FCDI) at a ratio of 6:1 were seeded at 8.49 × 10^4^ cells per well on 4-well and 24-well MEA plate (M384-tMEA-24W, Axion BioSystems). After 8 days of culture, the medium was replaced with BrainPhys Neuronal Medium with SM 1 neuronal supplement (STEMCELL technologies, United States). Half the media was exchanged every 4 days. Cell seeding was performed twice using different vials.

### Immunocytochemistry

Immunocytochemistry was performed using STAINperfect Immunostaining Kit A(SP-A-1000, ImmuSmol). The primary antibodies used for DA neurons (FCDI) were mouse anti- Tyrosine hydroxylase (ab129991, Abcam), rabbit anti-β -tubulin III (T2200, Sigma–Aldrich), chicken anti-GABA (IS1036, ImmuSmol), mouse anti-L-Glutamate (IS018, ImmuSmol), goat anti-FoxA2 Antibody (AF2400, R&D Systems), rabbit anti-dopamine D_1_ receptor antibody (ab20066, Abcam), and goat anti-dopamine D_2_ receptor antibody (ab30743, Abcam). Immunolabeling was visualized by incubation in an appropriate secondary antibody anti-mouse 488 Alexa Fluor (A-11001, Thermo Fisher Scientific), anti-rabbit 488 Alexa Fluor (A21206, Thermo Fisher Scientific), anti-mose 546 Alexa Fluor(A-10036, Thermo Fisher Scientific), anti-rabbit 546 Alexa Fluor (A-11010, Thermo Fisher Scientific), anti-chicken 647 Alexa Fluor (ab150175, Abcam), and anti-goat 680 Alexa Fluor (ab175776, Abcam), 1:1000 in Antibody Diluent (SP-A-1010, ImmuSmol) or Preblock buffer (0.05% Triton X and 5% goat serum in PBS) for 1 h at room temperature. The primary antibodies used for glutamatergic neurons (Gluta neuron) were mouse anti-L- Glutamate (IS018, ImmuSmol), chicken anti-GABA (IS1036, ImmuSmol). Anti-mouse 488 Alexa Fluor (A-11001, Thermo Fisher Scientific) and anti-chicken 546 Alexa Fluor (A-11040, Molecular Probes) were used as the secondary antibody. Cell nuclei of both DA neurons and glutamatergic neurons were counterstained using 1 μg/mL Hoechst 33258 (H341, DOJINDO) for 1 h at room temperature. The stained cells were observed under a fluorescent microscope (ECLIPSE TE2000-U, Nikon) and camera (DL-658M-OEN, Andor Technology), and image analysis was performed using LAS X Core (Leica).

### Extracellular Recording and Burst Analysis

Spontaneous extracellular field potentials were acquired at 37°C under a 5% CO_2_ atmosphere using a MEA system. Spontaneous firings of DA neurons were acquired using a 64-well MEA system (MED64-Allegro; Alpha Med Scientific) and 24-well MEA system (Presto; Alpha Med Scientific) at a sampling rate of 20 kHz/channel, and spontaneous firings of glutamatergic neurons were acquired using a 24-well MEA system (Maestro Edge; Axion BioSystems) at a sampling rate of 12.5 kHz/channel. Electrophysiological activity was first analyzed using Mobius software (Alpha Med Scientific) and MEA Symphony (Alpha Med Scientific) and AxIS software (Axion BioSystems) and MATLAB. A spike was counted when the extracellularly recorded signal exceeded a threshold of ± 5.3 σ, where σ was the standard deviation of the baseline noise during quiescent periods. SBFs were detected using the 4-step method (Matsuda et al., 2018), which was described previously. All data are expressed as the mean ± standard error (S.E.).

### Functional Evaluation of DA Neurons

To evaluate the electrophysiological function of cultured DA neurons, SKF 83822 hydrobromide, a D_1_ receptor agonist (SKF 83822; 0.1, 0.3, 1, 3, and 10 μM; 74115-10-9, R&D Systems; *n* = 6) and haloperidol (0.1, 0.3, 1, 3, and 10 μM; 084-04261, Wako; *n* = 5), a D_2_ receptor antagonist, were cumulatively administered, respectively. In addition, serotonin reuptake inhibitors sertraline hydrochloride (Sertraline; 0.1, 0.3, 1, 3, and 10 μM; 193-16191, Wako; *n* = 6) and paroxetine hydrochloride (Paroxetine; 0.3, 1, 3, 10, and 30 μM; PHR 1804-500 MG, Sigma; *n* = 6) were cumulatively administered, respectively. All compounds were dissolved in dimethyl sulfoxide (DMSO, 041-2351, Wako; *n* = 6) and diluted in culture medium. To confirm that the solvent DMSO did not affect the DA neuron activity, DMSO was cumulatively administered from 0.1 to 0.6% and the spontaneous activity was measured. Spontaneous activity was recorded for 10 min before and after administration of all drugs. The test was performed on a DN neuron network cultured in a 24-well MEA plate.

### Neurotransmitter Administration

To investigate dose-responses to neurotransmitters, we administered 5-Hydroxytryptamine Hydrochloride (Serotonin, 321-42341, Wako), Acetylcholine Chloride (Acetylcholine, 011-00592, Wako), Histamine (084-00643, Wako), DL-Norepinephrine hydrochloride crystalline (Noradrenaline, A7256-1G, sigma), Orexin A (Orexin, 159.03161, Wako), in cultured hiPSC-derived DA neurons on 4-well MEA plates, respectively. Neurotransmitters were cumulative administered to the culture medium at 5 concentrations (0.1, 1, 10, 100, and 1000 nM). Spontaneous firing was recorded for 10 min at each concentration. All neurotransmitters were administered dissolved in culture medium. The test was performed on cultured DA neuronal network in 4-well MEA plate (*n* = 4 well).

### Evocation of an Awake-Like State Using Serotonin

To induce a sleep–wake rhythm in a human iPSC-derived neural network, a long-term exposure test of serotonin was performed on DA neurons at the sixth week of culture. The study involved two conditions (culture medium alone and medium with 100 nM of serotonin added) that were alternately repeated three times in a 12-h cycle for a total spontaneous activity of 72 h, and the number of bursts and number of SBF under the two conditions were measured and compared.

### Induction of a Sleep-Like State via Low-Frequency Stimulation

We investigated whether a sleep-like state can be induced by administering electrical stimulation, which mimics the slow waves observed during non-REM sleep, to a human iPSC-derived neural network. Glutamatergic neurons at the 10th week of culture received a low-frequency stimulation (LFS), and changes in neural network activities before and after the stimulation were recorded. For the electrical stimulation, a rectangular wave of ± 20 μA with a maximum voltage of ± 1.2 V and duration of 0.2 ms was applied to all electrodes (16 ch/well) at 1 Hz.

### *Z*-Score

The connection strength of the neural network was assessed by the synchrony of activity between the electrodes (Kayama et al., 2018). If the firing interval between the two electrodes is <100 ms, these activities defined synchronized spike. These synchronized spikes were counted in spontaneous firings every 15 min before and after LFS. To determine how the number of synchronized spikes in real data differs from that when the firing is random, the *Z*-score was used. To use the synchronized spikes in the case of random firing as a population, surrogate data, in which the real interspike interval (ISI) obtained at each electrode was randomly shuffled, was created 100 times. The average number of synchronized spikes in 100 surrogate datasets was defined as *Ave*_surrogate_, and the standard deviation was *SD*_surrogate_. The *Z*-score was calculated based on the following equation, assuming that the number of synchronized spikes in real data without ISI replacement was defined as *N*_real_.

$$Z\;\mathrm{score}=\frac{N_{\mathrm{real}}-Ave_{\mathrm{surrogate}}}{{\mathrm{SD}}_{\mathrm{surrogate}}}$$

## Results

### Cultured hiPSC-Derived Neurons

**FIGURE 1:**
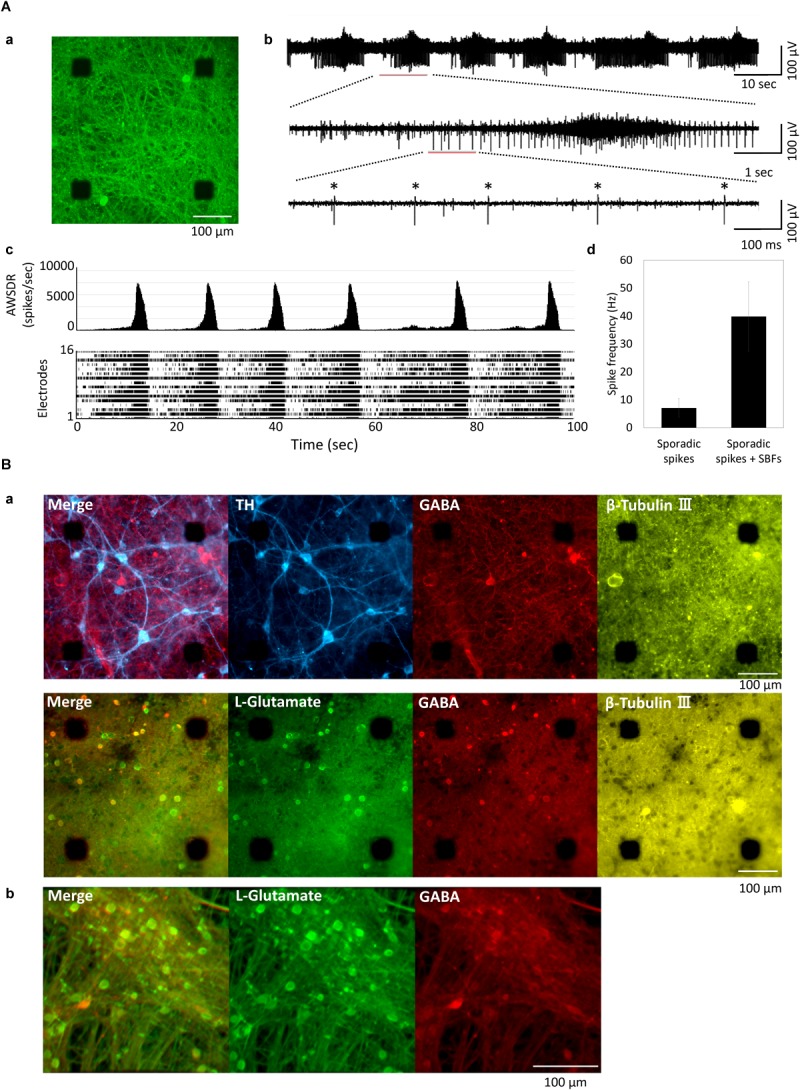
Cultured of human iPSC-derived neurons. **(A)** Human iPSC-derived neurons on micro-electrode array (MEA) chip and spontaneous activities. **(a)** Immunofluorescent image of human iPSC-derived neurons (iCell DopaNeurons) at 51 weeks *in vitro* (WIV). Images show the neurons using β-tubulin III immunostaining. Scale bars = 100 μm. **(b)** Typical waveform of spontaneous firings on different time scales for one electrode at 11 WIV. Asterisk (^∗^) indicates the firings of single neuron. **(c)** Array-wide spike detection rate (bin = 100 ms) and raster plots of spontaneous firing for 100 s at 16 electrodes per well. **(d)** Spike frequency of dopaminergic (DA) neuron. Spike frequency of spontaneous firings for 15 min was calculated for each electrode in cultured iCell DopaNeurons at 11 WIV (*n* = 16 electrodes/well, 4 wells). The analysis was divided into SBFs and non-SBF firings (sporadic spikes). In sporadic spikes, an electrode in which a spike was observed at 1 Hz or more was calculated as an active channel. **(B)** Immunofluorescence image of cultured human iPSC-derived neurons. **(a)** Immunofluorescence image of DA neurons on an MEA chip after 48 WIV. Tyrosine hydroxylase (TH, cyan), gamma-Aminobutyric acid (GABA, red), L-Glutamate (green), β-Tubulin (yellow). Scale bar = 100 μm. **(b)** Immunofluorescence image of glutamatergic neurons (iCell GlutaNeurons) on an MEA chip at 18 (WIV). Scale bar = 100 μm.

Figure 1Aa shows the human iPSC-derived neural network cultured on MEA. The action potential of the MEA-cultured neural network was measured using an extracellular recording method. Figure 1Ab depicts a typical spontaneous activity waveform for one channel. Figure 1Ac shows a histogram and raster plots of typical 16-channel neural network activity at 11 weeks *in vitro* (WIV). Synchronized burst firings (SBF) were first detected at 2 WIV and increased with culture time. Spikes were detected at all electrodes, and the frequency of SBF generation was 0.06 Hz (Figure 1Ac). The spike frequency was calculated for each electrode in the case of non-SBF firing (sporadic spikes) and total firings (sporadic spikes + SBFs) (Figure 1Ad). Sporadic spikes were 7.11 ± 3.39 Hz and sporadic spikes + SBFs were 39.8 ± 12.4 Hz. Immunochemical staining was performed to identify differentiated cells (Figure 1B). Cultured iCell DopaNeurons (DA neurons) at 23 weeks were stained using neuronal marker β-Tubulin III, cell nuclear marker Hoechst 33258, DA neuron marker tyrosine hydroxylase, and anti-GABA and anti-L-glutamate antibodies (Figure 1Ba). Cultured cells on the MEA were confirmed as the neurons by staining with the anti-β-Tubulin III antibody. The presence of DA neurons was also confirmed. However, GABAergic and glutamatergic neurons were also found in equal proportions. Furthermore, following 18 weeks of culture, iCell GlutaNeurons (Gluta neurons) were stained using anti-L-glutamate antibody, anti-GABA antibody, and Hoechst 33258. Subsequently, GABA neurons were determined to be a part of the neural network containing Gluta neurons (Figure 1Bb).

### Characterizations of DA Neuronal Network

**FIGURE 2:**
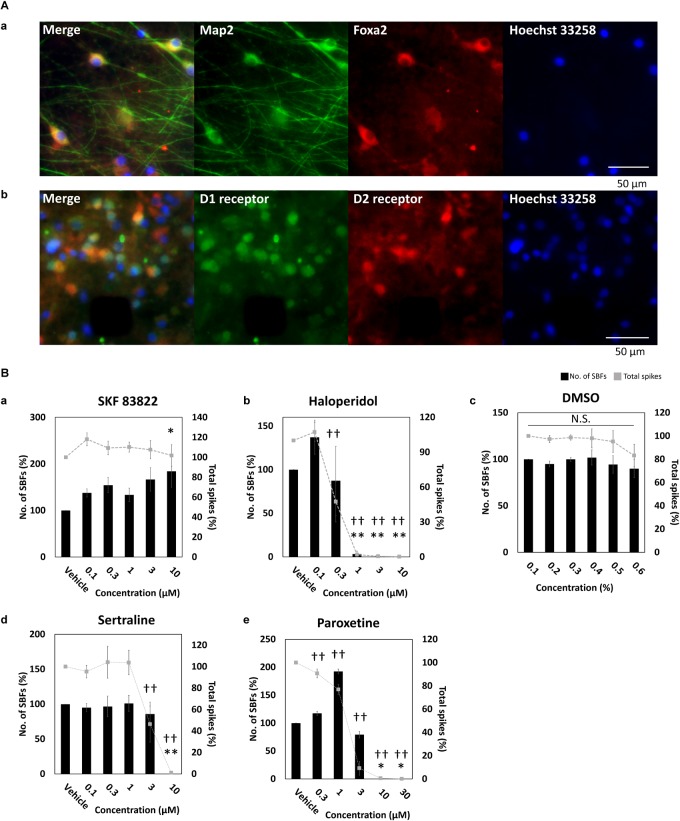
Characterization of dopaminergic neuron. **(A)** Immunofluorescent image of FOXA2, and D_1_ and D_2_ receptors in cultured iCell DopaNeurons at 23 WIV. **(a)** FOXA2 expression. MAP2 (green), FOXA2 (red), Hoechst 33258 (blue). Scale bars = 50 μm. **(b)** Dopamine receptor expression. D_1_ receptor (green), D_2_ receptor (red), Hoechst 33258 (blue). Scale bars = 50 μm. **(B)** The change of SBFs and spikes in SKF83822, haloperidol, DMSO, sertraline, and paroxetine administration. Spontaneous activity for 10 min was measured for each concentration. Black bars indicate SBFs and gray dashed lines indicate spikes. Data were analyzed using one-way ANOVA followed by *post hoc* Dunnett’s test [^∗^*p* < 0.05, ^∗∗^*p* < 0.01 vs. vehicle (SBFs), ^†^*p* < 0.05, ^††^*p* < 0.01 vs. vehicle (total spikes)]. **(a)** SKF 83822 (*n* = 6 wells). **(b)** Haloperidol (*n* = 5). **(c)** DMSO (*n* = 6). **(d)** Sertraline (*n* = 6). **(e)** Paroxetine (*n* = 6).

To identify midbrain DA neurons, FOXA2 (Ferri et al., 2007; Kittappa et al., 2007; Ang, 2009; Stott et al., 2013), a transcription factor important for development and maintenance of midbrain DA neurons, was stained and its expression was observed (Figure 2A). In addition to FOXA2 and LMX1A expressions, OTX2 expression, specifically present in ventral tegmental area (VTA) DA neurons (Di Salvio et al., 2010), has been also confirmed in the vendor data (FCDI) (data not shown). Cultured DA neurons in this experiment are also considered to contain midbrain VTA DA neurons. D_1_ and D_2_ receptor expressions was observed; however, not in all neurons (Figure 2A). Next, to confirm the electrophysiological function of the dopamine receptor, SKF 83822, a D_1_ receptor agonist, and haloperidol, a D_2_ receptor antagonist, were administered. SKF 83822 increased the number of SBF to 184 ± 34.3% at 10 μM (*p* = 0.0227, Figure 2Ba), whereas haloperidol increased the SBF to 137 ± 19.6% at 0.1 μM, decreased it to 3.33 ± 3.33% at high concentration 1 μM, and caused it to disappear at 3 μM (Figure 2Bb). Haloperidol is known to inhibit D_2_ and 5-HT2 receptors at low and high doses, respectively (Tyler et al., 2017). No change due to cumulative administration of DMSO was also confirmed (Figure 2Bc). Next, we investigated the function of DA neurons by administering sertraline and paroxetine, both of which are known to reduce the activity of VTA DA neurons via 5-HT2c receptors (Prisco et al., 1994; Di Mascio et al., 1998; Di Matteo et al., 2000). For both sertraline and paroxetine, SBFs disappeared at 10 μM (*p* = 0.001, Figure 2Bd,e). The increase in SBF by administration of SKF 83822 and 0.1 μM haloperidol indicates D_1_ and D_2_ receptors were functional. Functional 5-HT2c receptor expression in DA neurons was confirmed administration of sertraline and paroxetine.

### Enhancement of Neural Network Activity Through Administration of Neurotransmitters

**FIGURE 3:**
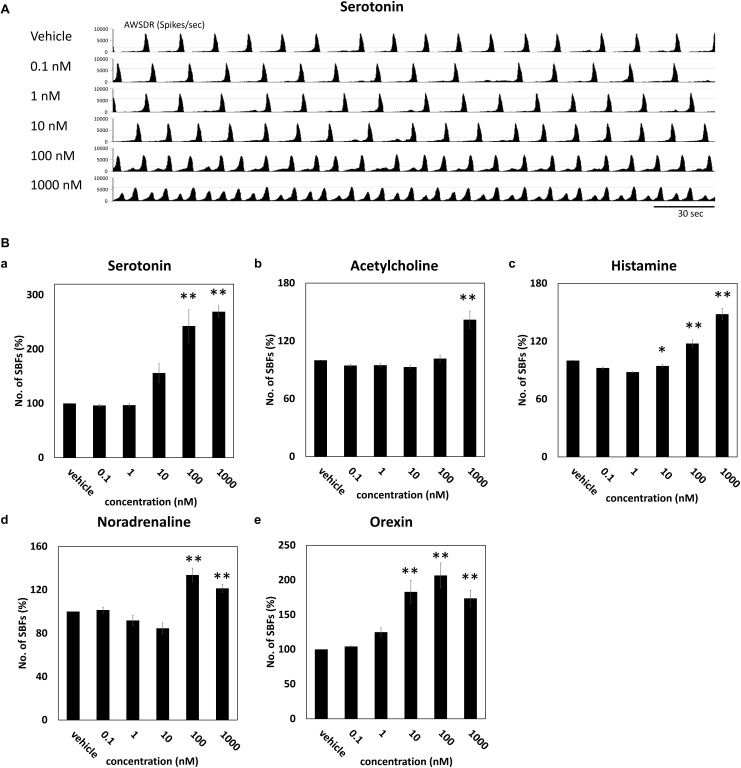
Neurotransmitter induced an increase of SBFs in human iPSC-derived dopaminergic neurons. Neurotransmitters were added to the culture medium at increasing concentrations (0.1, 1, 10, 100, and 1000 μM). Spontaneous firings were measured for 10 min before and after medication administration. **(A)** Typical array-wide spike detection rate of spontaneous firings for 5 min vehicle and serotonin administration. Scale bars = 30 s. **(B)** Changes in the number of SBFs versus vehicle (%) (*n* = 4 wells, One-way ANOVA and Holm test, ^∗^*p* < 0.05, ^∗∗^*p* < 0.01 vs. vehicle). **(a)** Serotonin. **(b)** Acetylcholine. **(c)** Histamine. **(d)** Noradrenaline. **(e)** Orexin.

Neurotransmitters known to be involved in the sleep–wake rhythm (serotonin, acetylcholine, histamine, noradrenaline, and orexin) were cumulatively administered to the cultured DA neuronal network to investigate their dose-dependent influence on the number of SBF (*n* = 4 wells). Figure 3A indicates the neural network activity at the time serotonin was administered. Following the administration of 100 nM serotonin, SBF markedly increased by 243 ± 29.7% (*p* = 0.001, Figure 3Ba). After the addition of 1000 nM acetylcholine, SBF increased by 142 ± 8.94% (*p* = 0.0001, Figure 3Bb). Following the administration of 100 nM histamine, SBF increased by 118 ± 3.71% (*p* = 0.00218, Figure 3Bc). After the addition of 100 nM noradrenaline, SBF markedly increased by 134 ± 6.22% (*p* = 0.001, Figure 3Bd). Finally, following the administration of 10 nM orexin, SBF markedly increased by 183 ± 16.9% (*p* = 0.001, Figure 3Be). From the above data, it was determined that receptors for each neurotransmitter are present in the human iPSC-derived neural network cultured in this study and, as the concentration of each neurotransmitter was increased, the synchronous activity of the network was enhanced.

### Evoking an Awake-Like Rhythm Through the Periodic Administration of Neurotransmitter

**FIGURE 4:**
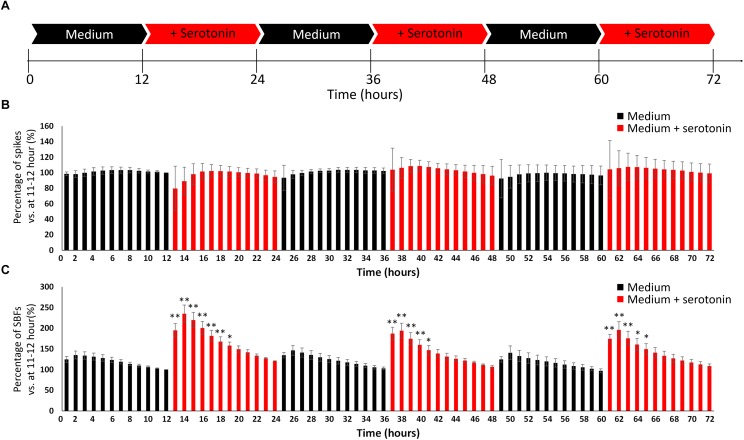
Evoking an awake-like rhythm through the periodic administration of serotonin. **(A)** Illustration of experimental scheme. The medium in cultured dopaminergic neurons was replaced with medium only (black arrows) or medium and serotonin (100 nM, red arrows) every 12 h. Two conditions were alternately repeated three times in a 12-h cycle, and spontaneous firings were measured for 72 h. Spontaneous firings were analyzed for every 1 h. **(B)** Changes in the number of spikes versus 11–12 h (%). **(C)** Changes in the number of SBFs versus 11–12 h (%). (*n* = 3, N is the number of wells and is the result of different culture samples. One-way ANOVA and *post hoc* Dunnett’s test, ^∗^*p* < 0.05, ^∗∗^*p* < 0.01 vs. 11–12 h). Black bars show the data for medium only, and red bars show the data for medium and serotonin (100 nM) condition.

We attempted to create the awake state in the cultured neural network by invoking the phenomenon that human iPSC-derived neural network activity is enhanced by neurotransmitters in the same way as this activity is boosted in the living brain. As illustrated in Figure 4A, this study included two experimental conditions (culture medium and culture medium with 100 nM serotonin) that were alternately repeated three times in a 12-h cycle for a total of 72 h, during which the number of spikes and SBF under the two conditions were compared. The number of spikes and SBF were calculated each hour and compared with numbers measured during serotonin administration. The values an hour immediately before the first addition of serotonin were set as 100% (one-way ANOVA and Dunnett’s test, Figure 4B). There was no significant difference in the spike rates between the two conditions (Figure 4B, *p* > 0.05, *n* = 3). However, the number of SBF markedly increased for 5 h after the administration of serotonin (Figure 4B, *p* < 0.05, *n* = 3) and then decreased with time. This experiment is the result of using another well than the one presented Figure 3B. The increase in SBFs was similarly observed after serotonin administration of serotonin, which indicates robustness. These rhythmic fluctuations in SBF within the 24-h cycle were repeatedly seen throughout the period during which serotonin was administered. This finding suggests that administering serotonin can produce a 24-h rhythmic cycle that mimics the awake state in the neural network.

### Changes in Spontaneous Activity via Low-Frequency Stimulation Mimicking the Slow Waves Generated During Sleep

**FIGURE 5:**
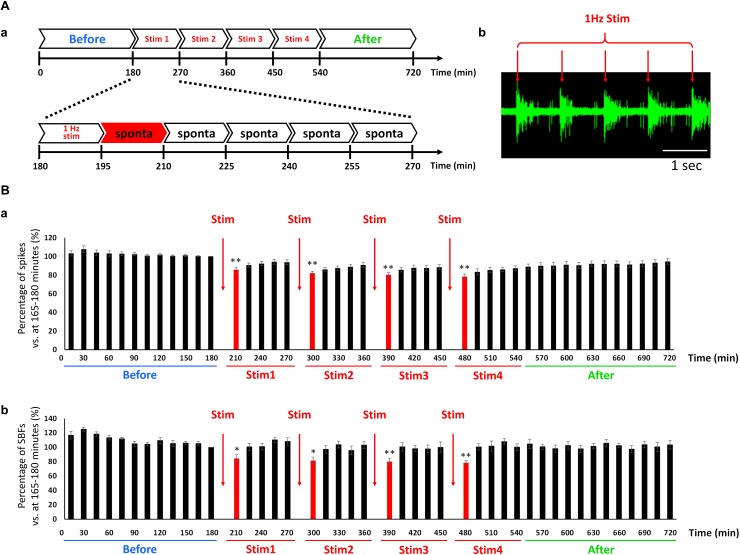
Change in the spontaneous firings via low frequency electrical stimulation (LFS) in cultured hiPSC-derived glutamatergic neurons. **(A)** Outline of experiment. **(a)** First, spontaneous activity was measured for 3 h before feeding the electrical stimulation (blue: before). The stimulus set (single set = 90 min) was subsequently fed to the network four times (red: Stim 1–4). The first 15 min of each set entailed electrical stimulation at 1 Hz and the remaining 75 min entailed the measurement of spontaneous activity. After performing four sets of stimulations (Stim 1–4), spontaneous activity was measured for 3 h (green: after). **(b)** Typical evoked responses during LFS. Scale bar = 1 s. Red arrow shows stimulus time and stimulation artifacts. **(B)** Change in the spontaneous firings after LFS at 6 WIV (*n* = 8). Black bars show the data for every 15 min, and red bars show the data for 15 min immediately after LFS. **(a)** Number of spikes vs. 165–180 min (%). **(b)** Number of SBFs vs. 165–180 min (%). Data were analyzed using one-way ANOVA followed by *post hoc* Dunnett’s test (^∗^*p* < 0.05, ^∗∗^*p* < 0.01 vs. before).

We examined whether a sleep state could be induced in a cultured human neural network by externally feeding 1-Hz electrical stimulation simulating the slow waves seen during sleep. The conceptual diagram of the experiment is shown in Figure 5Aa. Responses of the cultured human iPSC-derived neural network to 1 Hz electrical stimulation were observed (Figure 5Ab). The study confirmed that the neural network exhibited an evoked response to electrical stimulation at 1 Hz. The evoked response following electrical stimulation was confirmed for the fourth electrical stimulation (Supplementary Figure S1). Next, the number of spikes and SBF number before and after electrical stimulation were compared. The data from the 15 min during electrical stimulation were removed, and only the spontaneous activity data during the 15 min before and after stimulation were analyzed. Changes in the total number of spikes during those 15 min are shown in Figure 5Ba (*n* = 8). The total number of spikes was calculated by setting the data from 165 to 180 min before stimulation as 100%; data 15 min immediately after electric stimulation are indicated in red. The number of spikes during the 15 min directly after electrical stimulation decreased to the following levels after stim 1: 85.7 ± 2.74% (195–210 min), 82.1 ± 2.06% (285–300 min), 80.3 ± 2.52% (375–390 min), and 78.4 ± 2.77% (465–480 min) (*p* < 0.01, one-way ANOVA and Dunnett’s test). Furthermore, 4 h after stim 4 (after, 690–705 min), the number of spikes recovered to the state just before stimulation (before, 165–180 min) (93.3 ± 3.23%, *p* = 0.0756, *t*-test). Changes in SBF number during the 15 min is shown in Figure 5Bb. Similar to how the total number of spikes was calculated, the SBF number was calculated by setting the data from 165 to 180 min before stimulation as 100%; the data 15 min immediately after electric stimulation are indicated in red. The SBF number during the 15 min directly after electrical stimulation, which is indicated in red, sequentially decreased to the following levels directly after stim 1: 84.5 ± 5.05% (195–210 min), 81.6 ± 4.86% (285–300 min), 80.1 ± 4.81% (375–390 min), and 78.5 ± 3.47% (465–480 min) (*p* < 0.05, one-way ANOVA and Dunnett’s test). Furthermore, 30 min following stim 4 (after, 480–495 min), it recovered to the state prior to stimulation (before, 165–180 min) (101 ± 4.37%, *p* = 0.899, *t*-test). Based on these results, the human iPSC-derived neural network was found to evoke a sequential response to LFS mimicking slow waves during sleep. During the 15 min LFS, we were able to repeatedly reproduce the phenomenon of decreased frequency of spontaneous activity and recovery over time.

### Reduction of Synaptic Binding Strength via LFS

**FIGURE 6:**
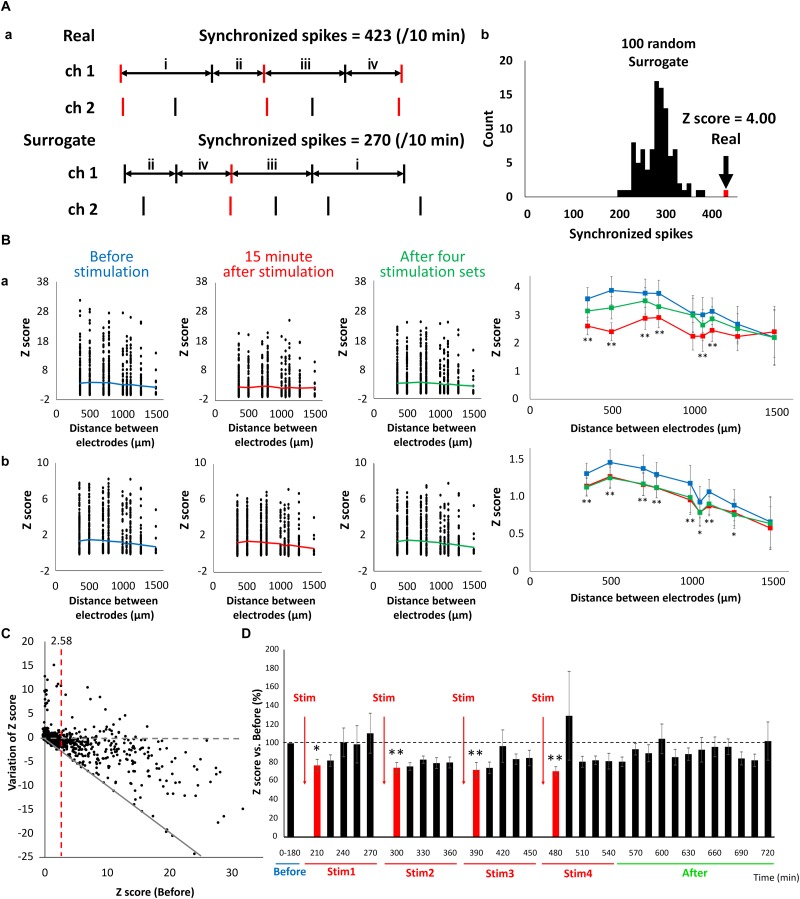
Reduction of connection strength in neuronal network caused by LFS. **(A)** Count of synchronized spikes between two electrodes. The number of synchronized spikes for every 15 min was counted within a 100 ms spikes (red). **(a)** Typical spike patterns of two electrodes (top). Each vertical line represents each spike. Typical surrogate dataset was generated by transposing the interspike intervals (ISIs) at random within an electrode (bottom). **(b)** Histogram of number of synchronized spikes. A real synchronized spikes is indicated by the arrow, which was compared with the distribution of synchronized spikes computed from the corresponding 100 surrogate datasets (black histogram). Based on the distribution of the surrogate datasets, the *Z*-score of the real data was computed to be 4.00. **(B)** The distribution of *Z*-score for each electrode distance (*n* = 120 pairs/well × 8 well = 960). The black plot shows the *Z*-score of each pair of electrodes; the polygonal line shows its average; **(a)** shows the data of sporadic firings that are not synchronized (sporadic); and **(b)** shows the data of synchronized bursts (burst) (blue; before, red; 15 min immediately after LFS, green; after 4 stimulation sets, *n* = 8). Right graphs show the average of *Z*-score before, 15 min immediately after LFS and after 4 stimulation sets in sporadic firings (upper) and burst firings (lower), respectively (two-tailed paired *t*-test, ^∗^*p* < 0.05, ^∗∗^*p* < 0.01 vs. before stimulation). **(C)** Distribution of variation of *Z*-score in sporadic spikes before and after LFS (*n* = 120 pairs/well × 8 well = 960). Variation value of *Z*-score defined *Z*-score (15 min immediately after LFS)—*Z*-score (before LFS). Gray dashed line shows variation of *Z*-score was zero (not affected by LFS), and gray line shows *Z*-score became zero after LFS. Red line indicates *Z*-score (before stimulation) = 2.58, which represents a significance level of *P* < 0.05. **(D)** Change in the *Z*-score versus before (%) (sporadic spikes, *n* = 8). Black bars show *Z*-score for every 15 min, and red bars show *Z*-score for 15 min immediately after LFS. Data were analyzed using one-way ANOVA followed by *post hoc* Dunnett’s test (^∗^*p* < 0.05, ^∗∗^*p* < 0.01 vs. before).

Changes in synaptic binding strength caused by LFS were evaluated based on the synchronism of electrical activity between the electrodes. The number of synchronized spikes between the two electrodes were counted based on 15-min spike data. A synchronized spike was defined as two spikes composed of one spike occurring within 100 ms of another spike occurring in the counterpart electrode (Figure 6Aa, top). Because synchronized spikes were dependent on the number of firings, we evaluated the strength of network activities using the *Z*-score of synchronized spikes. To quantify the synchronized spikes, 100 surrogate datasets composed of randomly switched ISI of each electrode were created, and the number of synchronized spikes was counted in the same manner (Figure 6Aa, bottom). The *Z*-score of the synchronized spikes of the actual spike data was calculated from the mean and standard deviation of the synchronized spikes obtained from the 100 surrogate datasets (Figure 6Ab). Figure 6B shows the distribution of *Z*-scores for each electrode distance, and indicates that *Z*-scores decreased after LFS and the binding strength of the neural network also decreased. Thus, the lesser the distance between the electrodes, the more profoundly the *Z*-score decreased 15 min immediately after stimulation. At the least distance of 350 μm between electrodes, the sporadic category decreased from 3.58 ± 0.401 to 2.61 ± 0.311, whereas the burst category significantly decreased from 1.31 ± 0.139 to 1.14 ± 0.120 (Figure 6Ba,b, right, *t*-test, *p* < 0.01). Figure 6C (*n* = 8 wells × 120 pairs/well = 960) illustrates the variation in the post-stimulation *Z*-scores with respect to the pre-stimulation *Z*-scores of the sporadic category. The dashed gray line in Figure 6C shows that the *Z*-score fluctuation was 0, whereas the red dashed line shows a pre-stimulation *Z*-score of 2.58. The solid gray line shows the proportional function with a slope of −1. As shown in Figure 6C, there were 321 pairs of electrodes with *Z*-scores (before) > 2.58 (*p* < 0.01), of which 267 pairs had a reduced *Z*-score from LFS, resulting in a *Z*-score reduction rate of 85.6% [(267/321) × 100%]. By contrast, there were 648 pairs of electrodes with *Z*-scores < 2.58, of which 345 pairs had reduced *Z*-scores due to LFS, resulting in a *Z*-score reduction rate of 53.2% [(345/648) × 100%]. These results show that the higher the pre-stimulation Z-score (or the stronger the binding strength) is, the greater the reduction. In addition, all pre-stimulation *Z*-scores ≥ 16.4 showed a decreased binding strength due to electrical stimulation. Therefore, using sporadic data, the temporal changes in *Z*-scores with respect to LFS were evaluated by analyzing only the electrode pairs with an average *Z*-score (before) > 2.58. The *Z*-score before LFS was set to 100%, and the average *Z*-score per 15 min after LFS was calculated (Figure 6D). The *Z*-score 15 min after LFS significantly decreased, starting with stim 1, in a sequential order to 76.4 ± 6.53, 74.0 ± 5.43, 71.7 ± 7.86, and 70.3 ± 4.77% (Figure 6D). LFS mimicking the slow waves seen during non-REM sleep tended to weaken the synaptic strength in the neural network, i.e., it weakened the excitability of the network.

## Discussion

As discussed, it is believed that many neurons in the wide modulating system play an important role in controlling the sleep–wake cycle. Recent studies have clarified that the acetylcholine, noradrenaline, histamine, serotonin, and orexinergic neurons administered in the present study are active during an awake state, compared to non-REM sleep (Mcginty and Harper, 1976; Takahashi et al., 2006, 2008, 2009, 2010; Sakai, 2012), and play critical roles in inducing an awake state (Adamantidis et al., 2007; Carter et al., 2010; Han et al., 2014; Van Dort et al., 2015). The present study entailed the administration of drugs to simulate modulation dependent on the sleep–wake state induced by these wide modulating system neurons. The results showed that, for all administered drugs, the number of synchronized bursts in the neural network increased (i.e., neural activity was activated). These results are consistent with those of the aforementioned *in vivo* animal experiments, indicating that the administration of a neurotransmitter can simulate an awake state in a cultured neural network.

The cultured cells used in this experiment include DA, glutamatergic, and GABAergic neurons (Figure 1Ba). DA neurons are present throughout the central nervous system but are especially localized in the substantia nigra and the ventral tegmental area (VTA) (Charles and Nemeroff, 2004). The expression of FOXA2, which is one of the important transcription factors for development and maintenance of midbrain DA neurons, was confirmed in the cultured neurons of this study (Figure 2A). In the vendor data, FOXA2 and OTX2 expressions, which are specifically observed in VTA DA neurons, are shown (data not shown). In addition, it has been shown in animal experiments that dopamine neurons often exhibit tonic firings, but sometimes burst firings. *In vivo* animal experiments have shown that VTA dopamine neurons tend to be in burst firings more than the substantia nigra dopamine neurons (Grenhoff et al., 1988; Zhang et al., 2008). Many of the neurons recorded in this study were burst firing, and neurons with tonic firing above 5 Hz were also observed (Figure 1Ab). In animal studies, serotonin reuptake inhibitors, sertraline and paroxetine, reportedly suppress VTA dopamine neuron activity via 5-HT2c receptors (Di Mascio et al., 1998). When sertraline or paroxetine is administered, VTA DA neuron activity reportedly decreases, but substantia nigra DA neuron activity does not (Di Mascio et al., 1998). In this study, DA neuron activity was attenuated with both sertraline and paroxetine. From these results, it can be concluded that the DA neurons used in this study include VTA DA neurons. On the other hand, to investigate the characteristics of VTA neurons in detail, it will be necessary to increase the efficiency of differentiation into VTA neurons. In addition, because it was difficult to distinguish cell-specific activities by spike sorting from this data, establishment of a spike sorting method is also important.

Since dopamine neurons do not change their bursting frequency depending on sleep–wake state, unlike other monoamine neurons, their role has not been regarded as important (Steinfels et al., 1983; Trulson and Preussler, 1984). However, recent work has shed light on the fact that VTA DA neurons that burst neural projections on the nucleus accumbens play a role in the induction and maintenance of an awakened state (Eban-Rothschild et al., 2016; Oishi et al., 2017). The VTA is composed of neural projections from the dorsal raphe nucleus (where serotonin neurons are found), locus coeruleus (where noradrenaline neurons are found), pedunculopontine tegmental area (where choline neurons are found), and lateral hypothalamus (where orexinergic neurons are found) (Oakman et al., 1995; Herbert et al., 1997; Fadel and Deutch, 2002). *In vivo* animal experiments have also shown that serotonin, acetylcholine, and orexin input to the VTA increases dopamine release (Guan and Mcbride, 1989; Nisell et al., 1994; Vittoz and Berridge, 2006). The concentration of serotonin in the hippocampus, hypothalamus, and prefrontal cortex is higher in an awake state than in a sleep state (Wilkinson et al., 1991; Park et al., 1999). Therefore, 100 nM of serotonin, which predominantly increases the number of synchronized bursts in the human iPSC-derived neural network, was administered in a 24-h cycle to a neural network containing dopamine neurons. Although there were no significant changes in the spike rate, the number of synchronized bursts increased during serotonin exposure. Further, change was seen in the number of synchronized bursts within a 24-h cycle. It is known that DA neurons do not change their number of spikes depending on the sleep–wake state (Steinfels et al., 1983; Trulson and Preussler, 1984); however, the number of synchronized bursts increase during an awakened state and REM sleep (Dahan et al., 2007). Therefore, the same phenomenon as observed *in vivo* nerve activity was generated in cultured cells lacking the structures seen in a living body by externally administering drugs. The increase in synchronized bursts with serotonin administration decreased with time, ultimately decreasing to the rate before administration at 6 h after serotonin administration. In a living brain, serotonin is metabolized into 5-hydroxyindoleacetic acid by monoamine oxidase (Sjoerdsma et al., 1955). The serotonin concentration in the hypothalamus and prefrontal cortex of rats reaches its peak during the day, when it is the most active, and reaches its lowest level approximately 5.5 h later (Quay, 1968). Although temporal changes in the serotonin concentration of the neural network in the present study are unknown, temporal changes in the number of synchronized bursts after serotonin administration may be related to reduced serotonin levels due to the metabolism of serotonin, as observed *in vivo*.

Sleep is thought to play a role in maintaining body function by saving energy and regulating body temperature (Mcginty and Szymusiak, 1990; Berger and Phillips, 1995). In recent years, it has been investigated whether sleep is involved in maintaining brain function, and it has been suggested that sleep contributes to the maintenance of neural circuits (Diekelmann and Born, 2010). The synaptic homeostasis hypothesis proposed by Tononi and Cirelli (2003) is regarded as a powerful model of sleep’s mechanism and function. In this hypothesis, the increase in the synaptic strength of the cerebral neocortex at the time of awakening is considered to be uniformly attenuated during sleep such that synaptic strength is presumed to be maintained within a certain range. Evidence that synaptic connective strength is attenuated during sleep has recently been reported (Vyazovskiy et al., 2008; Liu et al., 2010; Huber et al., 2013; De Vivo et al., 2017), but it has not yet been confirmed whether it is uniformly attenuated. In the present study, we focused on the slow waves generated during non-REM sleep and fed low-frequency electrical stimulation to the network to simulate non-REM sleep. Because slow waves occur in a 90 min cycle in the deep non-REM sleep (Keenan and Hirshkowitz, 2011), electrical stimulation was repeated every 90 min. The report of Mukovski et al. (2007), which simultaneously recorded the intracellular activity and local field potential (LFP) in cats, suggested that the LFP is the negative amplitude in the up state where the intracellular potential is high and the neuron is active. Conversely, when the local field potential is the positive amplitude, the intracellular potential is low and neuronal activity ceases. In other words, the neural activity, intracellular potential, and extracellular field are in antiphase. Because the electrical stimulation used in this study is considered to have the function of activating the neural activity, it is considered that the 1 Hz electrical stimulation mimics the slow wave. Synaptic connective strength was quantified as a *Z*-score to illustrate the difference in synchronized spikes in activity between the two electrodes compared to the 100 surrogate datasets with randomly shuffled ISI. The *Z*-score representing transmission intensity decreased for a combination of some electrodes after LFS, whereas there were almost no combinations in which *Z*-score increased. This result suggests the reconstruction of the phenomenon in which synaptic binding decreases during sleep. This study also investigated changes in *Z*-score before and after LFS and found a trend of a greater decrease in *Z*-score after LFS for combinations of electrodes that originally had large *Z*-scores. This finding suggests that attenuation of synaptic connections was not uniform; rather, they were induced only in part (particularly in electrodes that originally had strong binding) despite applying stimulation at the same intensity to all electrodes. As shown in Figure 6C, in the circuit where the binding is strong before LFS, the probability that the synaptic connection is attenuated is high, suggesting that LFS made the strength of the connection uniform. Therefore, it is considered that the phenomenon caused by LFS may be called homeostasis.

To induce a sleep–wake-like state in a human iPSC-derived neural network cultured on MEA, we administered the relevant neurotransmitters and LFS mimicking the slow waves seen during sleep. Periodic neurotransmitter administration mimicked wake-like nerve activity, whereas LFS induced a decrease in nerve activity and network binding. These results suggest that it is possible to mimic sleep–wake states *in vitro* by externally stimulating a human iPSC-derived neural network. To simulate a real biological phenomenon as accurately as possible, considerations such as simulating circuit structure should be made. The findings of the present study can be applied in studies on the human sleep–wake regulation mechanism related to LTD or for an *in vitro* drug efficacy evaluation system exhibiting a circadian rhythm. If the differences between the iPSC-derived diseased neurons related to sleep and healthy neurons can be detected using this method and subsequent analysis, it can be applied to understand disease mechanisms and screen drugs for sleep disorders. In addition, the method in this study can be applied to assess the seizure liability of new drugs depending on sleep and wakefulness.

## Author Contributions

IS designed the research. RY and MO performed the experiments and analyzed all data. RY prepared all figures. NM analyzed the data in Figure 5. AO and AK discussed the analyzed data. RY, AK, and IS wrote the main manuscript text. All authors reviewed the manuscript.

## Conflict of Interest Statement

The authors declare that the research was conducted in the absence of any commercial or financial relationships that could be construed as a potential conflict of interest.
