# Supplementary material for: Impact of Sleep–Wake-Associated Neuromodulators and Repetitive Low-Frequency Stimulation on Human iPSC-Derived Neurons
Source: Front Neurosci. 2019 May 29;13:554. doi: 10.3389/fnins.2019.00554 (PMC6549533; doi:10.3389/fnins.2019.00554)
Supplement: Supplementary file 1 [file Data_Sheet_1.docx]

Supplementary Material

## Supplementary Figures


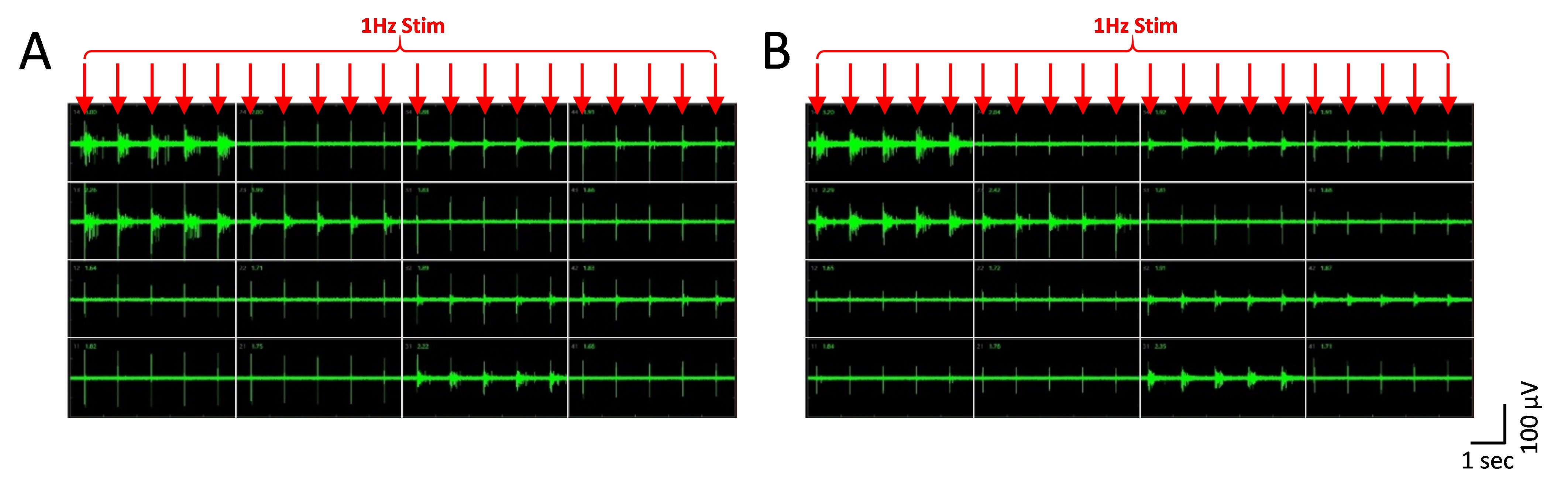


**Supplementary Figure 1.** Typical evoked responses in 16 electrodes following LFS in glutamatergic neuron. Red arrow shows stimulus time and stimulation artifacts. (A) Typical evoked responses following stimulation set 1 (Stim 1). (B) Typical evoked responses following stimulation set 4 (Stim 4). We confirmed evoked responses following both stimulations set 1 and 4. Further, evoked responses were confirmed in more than half of 16 electrodes.
